# Paternal Folate Status and Sperm Quality, Pregnancy Outcomes, and Epigenetics: A Systematic Review and Meta‐Analysis

**DOI:** 10.1002/mnfr.201900696

**Published:** 2020-02-20

**Authors:** Jeffrey Hoek, Régine P. M. Steegers‐Theunissen, Sten P. Willemsen, Sam Schoenmakers

**Affiliations:** ^1^ Department Obstetrics and Gynecology, Erasmus MC University Medical Center Rotterdam 3015GD The Netherlands; ^2^ Department Biostatistics, Erasmus MC University Medical Center Rotterdam 3015GD The Netherlands

**Keywords:** congenital malformations, epigenetics, fertility, folate, folic acid supplements, placenta, sperm quality

## Abstract

**Scope:**

The effectiveness of maternal folate in reducing the risk of congenital malformations during pregnancy is well established. However, the role of the paternal folate status is scarcely investigated. The aim of this study is to investigate the evidence of associations between the paternal folate status and sperm quality, sperm epigenome, and pregnancy outcomes.

**Methods and results:**

Databases are searched up to December 2017 resulting in 3682 articles, of which 23 are retrieved for full‐text assessment. Four out of thirteen human and two out of four animal studies show positive associations between folate concentrations and sperm parameters. An additional meta‐analysis of four randomized controlled trials in subfertile men shows that the sperm concentration increases (3.54 95% confidence interval (CI) [−1.40 to 8.48]) after 3–6 months of 5 mg folic acid use per day compared to controls. Moreover, two out of two animal and one out of three human studies show significant alterations in the overall methylation of the sperm epigenome. One animal and one human study show associations between low folate intake and an increased risk of congenital malformations.

**Conclusions:**

This systematic review and meta‐analysis shows evidence of associations between paternal folate status and sperm quality, fertility, congenital malformations, and placental weight.

## Graphical abstract

The aim of this study is to investigate and provide an overview of the evidence of associations between the periconceptional paternal folate status and folic acid supplement use and sperm quality, sperm epigenome, and pregnancy outcomes in both human and animal studies. It is concluded that more research is necessary into the periconceptional roles of paternal micronutrients.

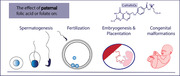

## 1 Introduction

**Figure 1:**
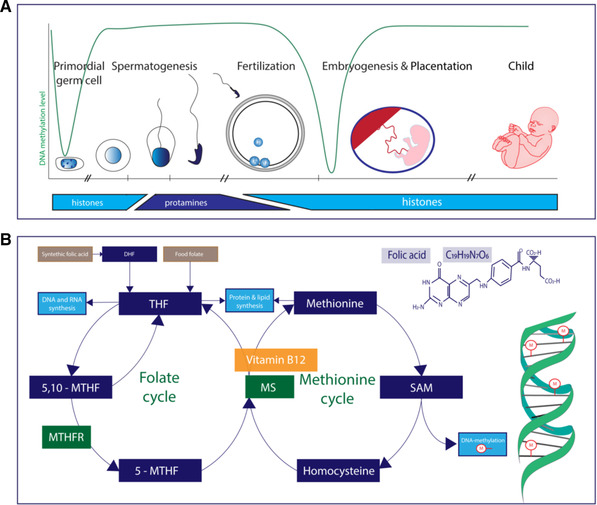
Overview of a) the spermatogenesis, embryogenesis, the corresponding histone‐protamine exchange, and the methylation level of non‐imprinted genes and b) folate related one‐carbon metabolism. DHF, dihydrofolate; THF, tetrahydrofolate; 5,10‐MTHF, 5,10‐methylenetetrahydrofolate; MTHFR, methylenetetrahydrofolate reductase; 5‐MTHF, 5‐methyltetrahydrofolate; MS, methionine synthase;SAM, S‐adenosylmethionine. Dark blue box: proteins; green box: enzymes; yellow box: vitamin/cofactor; light blue box: processes.

The last three decades of research have shown overwhelming evidence that the B vitamin folate is essential for reproduction, pregnancy, health, and disease. In preconception care, maternal folic acid (FA) supplement use is well known for its role in the prevention of congenital malformations, in particular, neural tube defects and congenital heart defects.^[^ ^1^ ^]^ Due to the proven protective role of FA in human reproduction, the World Health Organization advises all women to use 0.4 mg FA from the moment of contemplating pregnancy up to 12 weeks of gestation. Van Uitert et al. showed in a systematic review that red blood cell (RBC) folate concentrations and FA supplement use is positively associated with an increased birthweight and inversely associated with the risk of low birthweight and small for gestation age infants.^[^ ^2^ ^]^ These effects can be explained by impaired cell multiplication, DNA synthesis, and programming due to (ir) reversible changes of the epigenome, such as DNA methylation, histone modifications, and chromatin remodeling, induced during gametogenesis and the first weeks after conception. The periconceptional epigenome of both men and women, together with transcription factors, RNA and one‐carbon (1‐C) moieties play key roles in molecular biological processes, such as programming of gene expression, involved in embryonic, fetal, and placental growth and development (**Figure** **1**a).^[^ ^3^ ^]^

Folate, but also methionine and choline, are important substrates of the 1‐C metabolism, which provides essential 1‐C moieties for processes such as lipid, nucleotide, protein, and DNA synthesis, but also for methylation of DNA and histones.^[^ ^4^ ^]^ The main natural sources of folate are fruits, vegetables, and nuts, which are absorbed from the jejunum as the biological active form of tetrahydrofolate (THF). Another source is synthetic FA derived from fortified foods and supplements, that first need to be converted in the intestinal cells by dihydrofolate reductase (enzyme commission number [ECN]: 1.5.1.3) to the active form, THF. The next essential step is the conversion of THF into 5‐methyl‐tetrahydrofolate (5‐MTHF), by the enzyme methylenetetrahydrofolate reductase (MTHFR, ECN: 1.5.1.20). 5‐MTHF together with homocysteine is converted into methionine by methionine synthase (MS) (ECN: 2.1.1.13) using vitamin B12 as cofactor. The folate‐dependent 1‐C metabolism is necessary for the production of essential 1‐C moieties (Figure 1b). Single nucleotide polymorphisms (SNPs) in essential genes of the folate dependent 1‐C metabolism, such as *MTHFR* (ECN: 1.5.1.20), can affect enzymatic activities and the availability of 1‐C moieties. Altogether, differences in the intake of folate, FA, and individual SNPs, in tissues and target organs and the combination of all these factors greatly influence the availability of 1‐C moieties.

Since the embryo and fetus develop within the maternal environment, it is not surprising that previous research has mainly focused on the maternal folate status in relation to periconceptional and pregnancy outcomes. Although the father‐to‐be also contributes half of the genetic material to the offspring and the placenta, the periconceptional paternal folate status has hardly been investigated. This is surprising whereas it is known that paternal folate concentrations can affect sperm quality including its DNA integrity and epigenome.^[^ ^5^, ^6^, ^7^ ^]^ Therefore, we hypothesize that the paternal folate status could not only affect DNA methylation and sperm quality, but also fertility, and after successful conception, miscarriage risk, embryonic growth, fetal and placentation development, and pregnancy outcome.

Spermatogonial stem cells are present from birth but the process of spermatogenesis only takes place in ≈2–3 months. During spermatogenesis, millions of spermatozoa are produced per day, indicating that the production of proteins and DNA are needed on a large scale. In the human testes, the male germ cells develop into spermatids and eventually into spermatozoa (sperm), during spermiogenesis. The differentiation process of spermiogenesis consists of major morphological and chemical alterations and is necessary to ensure that the nuclear DNA will be tightly compacted in the spermatozoal head. The histone to protamine exchange, in which most histones are replaced by protamines, allows a more condensed chromatin structure allowing the tight formation of DNA (Figure 1a).^[^ ^8^ ^]^ Interestingly, retained histones with epigenetic information from the father can be transferred to the conceptus. Since spermatogenesis takes place in a relatively short time period, we hypothesize that paternal nutrition and lifestyle can have a relatively direct impact on reproductive success and pregnancy outcomes with short and long‐term health effects for the offspring. Herein, we aim to give an overview of the evidence on associations between the periconceptional paternal folate status and sperm quality, pregnancy outcomes, and epigenetics (Figure 1a).

## 2 Experimental Section

### 2.1 Search Strategy

Searches were performed in the databases of Embase, Medline, PubMed, Web of Science, the Cochrane databases, and Google Scholar. The protocol for this systematic review was designed and registered a priori at the PROSPERO registry (PROSPERO 2017: CRD42017080482). The search strategy terms used the following MeSH terms including but not limited to FA, folate, sperm, fertility, miscarriage, placenta, and pregnancy outcome (Table S2, Supporting Information). These were combined using the Boolean operator “or”.

### 2.2 Inclusion and Exclusion Criteria

#### 2.2.1 Systematic Review Eligibility Criteria

The paternal folate status was defined as folate concentrations measured in blood or seminal plasma. Determinants of folate status included in the search are intake of FA, folate intake, and 1‐C metabolism.

The main outcomes are divided in preconceptional and postconceptional outcomes. The preconceptional outcomes consisted of sperm parameters (sperm concentration, sperm count), sperm DNA damage, and sperm DNA‐methylation. Fertility, time‐to‐pregnancy, miscarriage, fetal growth (small for gestational age, intra‐uterine growth restriction, and birthweight), placentation, and (preterm) birth were considered as postconceptional outcomes. Databases were searched up and till December 2017. The results of all the outcome searches were combined with “or”. The results of the paternal folate status and outcome searches were then combined with “and”.

Animal and human studies comprising experimental studies, observational cohorts, case control studies, and randomized controlled trials (RCTs) were eligible for inclusion in the review.

Letters, conference abstracts, editorials, and case reports were excluded and the search was restricted to English language papers.

Articles describing male participants with or without sperm dysfunction were included, as were papers investigating administration of high or low doses of FA compared to a control dose. Studies measuring folate concentrations in blood or seminal plasma as exposure variable were also included. Maternal only as well as combined paternal and maternal FA interventions were excluded.

#### 2.2.2 Study Selection, Full Text Review, and Data Extraction

J.H. and S.S. reviewed the titles and abstracts independently from each other and selected papers for the full‐text review. Next, full text reviewing and data extraction were also independently performed by J.H. and S.S. Data were put into a template, specifically for this review. Differences were resolved by discussion between these authors. Any disagreements concerning the eligibility of particular studies were resolved through discussion with a third reviewer (RST). Data extracted included the country of origin, year of publication, study design, study population (including human or animal), sample size, exposures of interest, outcome data, exclusion criteria, statistical analysis, potential confounders, results, and conclusion.

#### 2.2.3 Quality of Study and Risk of Bias

To assess the quality of the human studies included in the review, the ErasmusAGE quality score for systematic reviews was used: a tool composed of five items based on previously published scoring systems. Each of the five items can be allocated either zero, one, or two points giving a total score between zero and ten, with a score of ten representing a study of the highest quality. The five items include study design (0 = cross‐sectional study, 1 = longitudinal study, 2 = intervention study), study size (0 = <50, 1 = 50 to 150, 2 = >150 participants), method of measuring exposure (0 = not reported, 1 = moderate quality exposure, 2 = good quality exposure), method of measuring outcome (0 = no appropriate outcome reported, 1 = moderate outcome quality, 2 = adequate outcome quality), and analysis with adjustments (0 = no adjustments, 1 = controlled for key confounders, 2 = additional adjustments for confounders) (Table S1, Supporting Information).^[^ ^9^ ^]^

### 2.3 Meta‐Analysis

An additional meta‐analysis of only human data was conducted to investigate the effects of 5 mg FA per day supplement use for 3–6 months in subfertile males on sperm concentration, sperm motility, and normal sperm morphology. For the other outcomes considered in this systematic review, unfortunately, not enough information was available for meta‐analysis.

The difference‐in‐difference of three outcomes is extracted and pooled: sperm concentration, sperm motility, and normal sperm morphology. The difference‐in‐difference is the difference between the effects of the treatment in the intervention and the control group, where the effect of the treatment is measured as the difference between the outcome after and before the intervention. When no information was available of the effect on the outcomes, it was computed based on the published baseline and follow‐up measurements. When standard deviations were not given, they were calculated based on standard error and sample size or approximated using the interquartile range (using the assumption of normality). None of the studies published the standard error of the difference between the pre‐ and post‐intervention outcomes. To compute these, the estimates of the correlation were based between the two time points on the data of Wong et al. The pooling of effects was done using a random‐effects model estimated by restricted maximum likelihood and the heterogeneity was assessed using the *I* ^2^‐value. Pooled effects with a *p*‐value of 5% were considered significant. Any multiplicity adjustment was not applied.

## 3 Results and Discussion

### 3.1 Study Selection

**Figure 2:**
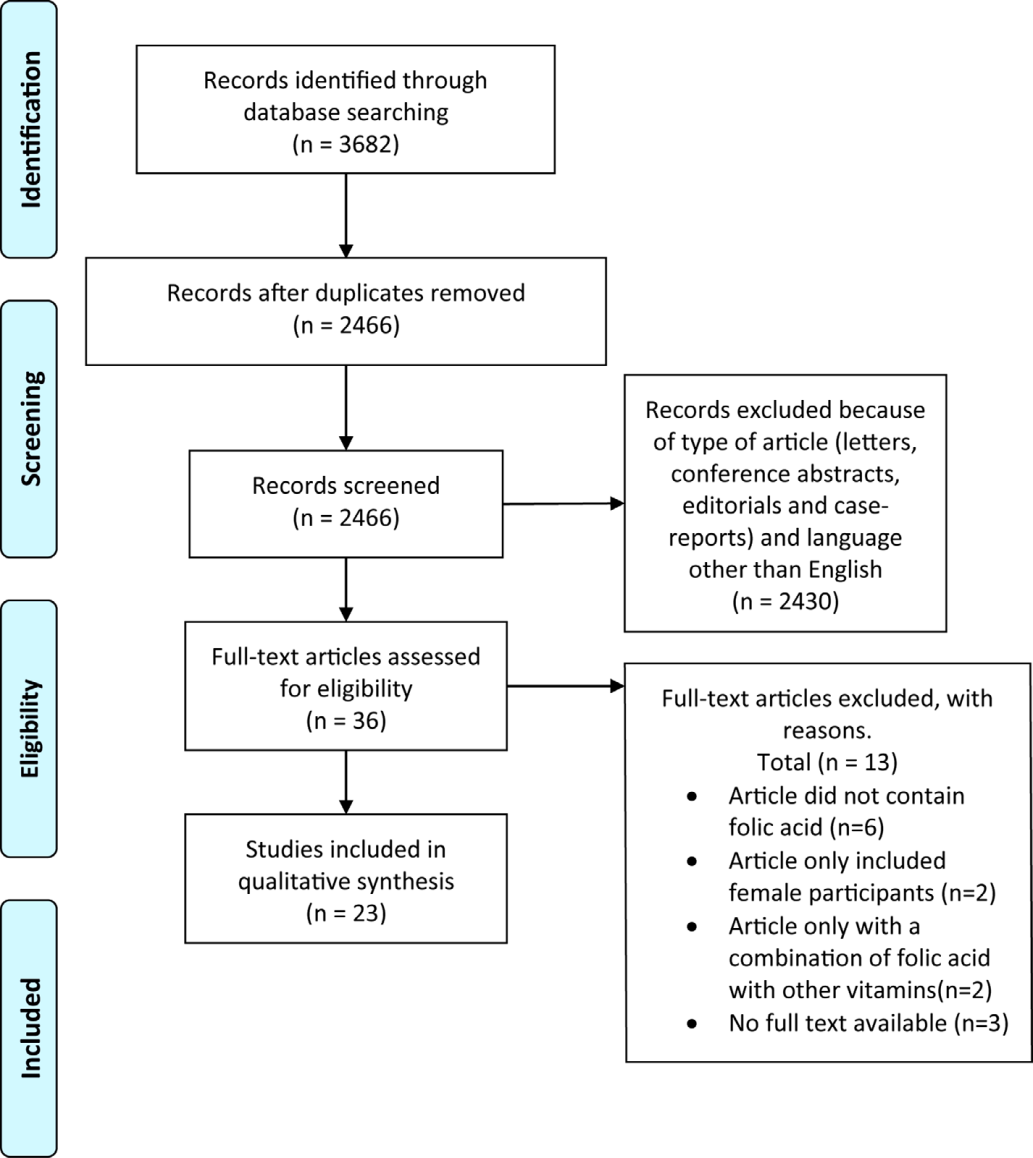
Flowchart of in‐ and excluded studies.

The flowchart summarizes the process of literature search and selection of studies (**Figure** **2**). The initial search identified 3682 records of which 1216 were duplicates. Of the remaining 2466 records, a total of 2430 publications were excluded because they did not fulfil the selection criteria. The full text of 36 papers were read, 13 papers were excluded, resulting in 23 remaining articles for analysis. The general characteristics of all included studies and all specific concentrations of FA supplemented/deficient animal diets are shown in **Table** **1**. Of these 23 articles, 6 are animal studies, 1 article combined a human and animal study, and 16 represent human studies, including 6 randomized controlled trials (RCTs), 4 case‐control studies, 3 cross‐sectional studies, 2 intervention studies, 1 pre‐post analysis, and 1 prospective cohort study.

**Table 1:** Main characteristics of 23 included studies

Author	Year	Country	Study population	Study design	Sample size	Exposure(s)	Outcome(s)	Quality score
Aarabi et al.	2015	Canada	Healthy normozoospermic men presenting with idiopathic infertility stratified for three types of *MTHFR* gene polymorphisms	Prospective intervention study	30	6 months high dose (5 mg) FA	Sperm quality according to WHO, sperm DNA damage, and sperm epigenetics	4
Boonyarangkul et al.	2015	Thailand	Men with abnormal sperm analysis	RCT	68	FA supplementation 5 mg per day for 3 months	Sperm quality according to WHO and sperm DNA‐damage	9
Boxmeer et al.	2007	The Netherlands	Fertile and subfertile men	Cross‐sectional	111	Folate concentrations in serum, RBC, and seminal plasma	Sperm quality according to WHO	7
Boxmeer et al.	2009	The Netherlands	Fertile and subfertile men	Cross‐sectional	279	Folate concentrations in serum, RBC, and seminal plasma	Sperm quality according to WHO and sperm DNA‐damage	8
Chan et al.	2017	Canada	Men exposed to FA food fortification for years	Pre–post study	27	Food fortification	Sperm epigenetics	4
Crha et al.	2010	Czech Republic	Men with azoospermia and normozoospermia controls	Cross‐sectional	134	Folate concentrations in serum and seminal plasma	Sperm quality according to WHO and testicular volume	5
Da Silva et al.	2013	Brazil	Subfertile men 20–55 years	RCT	70	FA supplementation 5 mg/day for 3 months	Sperm quality according to WHO	8
Ebbisch et al.	2006	The Netherlands	Fertile and subfertile men	RCT	87	Four groups: FA 5 mg, zinc 66 mg, zinc and FA and placebo for 26 weeks	Sperm quality according to WHO	4
Ebbisch et al.	2005	The Netherlands	Fertile and subfertile men	RCT	164	Four groups: FA 5 mg, zinc 66 mg, zinc and FA and placebo for 26 weeks	Sperm quality according to WHO and seminal annexin A5 (apoptosis marker)	9
Kim et al.	2011	Korea	Male rats on either a folate supplemented or folate deficient diet	Animal study	14	Folate deficient (0 mg) or folate rich (8 mg/kg diet) diet for 4 weeks	Fetal growth, fetal liver and placenta folate content. Folate receptor alfa expression	N/A
Kim et al.	2013	Korea	Male rats on either a folate supplemented or folate deficient diet	Animal study	12	Folate deficient (0 mg) or folate rich (8 mg kg^−1^ diet) diet for 4 weeks	Fetal growth, total folate content in fetal liver and brain and IGF2 expression in fetal brain	N/A
Lambrot et al.	2013	Canada	Male mice (C57BL/6) on either a folate supplemented or folate deficient diet already in utero exposed to the same feeding regime	Animal study	128	Folate deficient (0.3 mg kg^−1^) or folate rich (2 mg kg^−1^) diet	Sperm morphology, sperm epigenome, pregnancy rate, miscarriage, fetal growth and congenital malformations	N/A
Landau et al.	1978	Israel	Men with normo‐ and oligospermia	Prospective intervention study	40	FA 10 mg for 30 days	Sperm concentration and motility, DNA content of the spermatozoa	3
Ly et al.	2017	Canada	Male mice (BALB/c) already in utero exposed to four feeding regimens and during life fed the same regimen	Animal study	60	Four feeding regimens (control; 2 mg kg^−1^ FA, 0.3 mg kg^−1^) FA, 20 mg kg^−1^ FA and 40 mg kg^−1^ FA) from mothers and during life fed the same regimen.	Sperm count and DNA methylation, fetal placenta and brain DNA methylation and miscarriage	N/A
Mejos et al.	2013	Korea	Male and female rats who got folate supplemented or deficient diet	Animal study	40	Folate deficient (0 mg) or folate supplement (8 mg kg^−1^) diet for 4 weeks	Postnatal hepatic folate content and DNA methylation and hepatic FR alfa, IGF‐2, and IGF‐1R expression	N/A
Murphy et al.	2011	Sweden	Infertile men who are 20–45 year old, having regular sexual intercourse >1 year without a pregnancy. Fertile men: who are 20–45 year old and conceived at least 1 pregnancy who now stopped birth control	Case‐control study	337	Folate concentrations in serum and seminal plasma	Sperm quality according to WHO and SNP genotyping in genes related to folate metabolism	8
Pauwels et al.	2017	Belgium	Caucasian men	Prospective cohort study	51	Paternal methyl group intake	Paternal and offspring global DNA methylation and offspring IGF2 methylation and birthweight	8
Raigani et al.	2014	Iran	Subfertile oligoasthenoteratozoospermic men	RCT	83	Four groups: FA 5 mg, zinc 220 mg, zinc and FA and placebo for 16 weeks	Sperm quality according to WHO and sperm DNA damage	9
Ratan et al.	2008	India	Neonates with neural tube defects as cases. Controls: neonates with other congenital malformations and neonates with no abnormalities	Case‐control study	90	Serum and RBC folate concentrations	congenital malformations	3
Swayne et al.	2012	Canada	Male mice (BALB/c) were given control, folate deficient or folate supplemented diet already started in utero and switched to control diet during weaning	Animal study	96	Control diet with FA 2 mg kg^−1^. FA deficient diet contained 0 mg kg^−1^ FA and FA supplemented contained 6 mg kg^−1^ FA	Cauda epididymal sperm counts and sperm DNA damage	N/A
Wallock et al.	2001	USA	Healthy male smokers and non‐smoker with a low intake of vegetables and fruit, aged 20–50 years	Case‐control study	48	Serum and seminal folate concentrations	Sperm count and density	6
Wong et al.	2002	The Netherlands	Fertile men: no history of fertility problems and a current pregnant partner. Subfertile men: failure to conceive after 1 year of unprotected intercourse and a sperm count between 5 and 20 million per mL.	RCT	194	Four groups: FA 5 mg, zinc 66 mg, zinc and FA and placebo for 26 weeks	Sperm quality according to WHO	9
Yuan et al.	2017	China	Human: male subfertile patients aged 18–55 years with azoospermia and normospermia. Animals: female mice (C57BL/6) were given a folate deficient diet or control diet already started in uteruo and male offspring were fed the same regimen	Case‐control study and animal study	269	Human: seminal folate concentrations Animal: FA deficient (0.3 mg kg^−1^) diet and control diet	Human: sperm quality according to WHO, DNA methylation and protein expression Animal: sperm counts, testis histology, and proteins	8

### 3.2 Preconceptional

A total of four studies investigated the associations between folate status and sperm parameters in animals.^[^ ^10^, ^11^, ^12^, ^13^ ^]^ Furthermore, 13 articles reported on the association between folate status and sperm parameters in human.^[^ ^5^, ^6^, ^7^, ^12^, ^14^, ^15^, ^16^, ^17^, ^18^, ^19^, ^20^, ^21^, ^22^ ^]^ A total of five studies investigated the association between folate status and sperm epigenetics, of which two were animal studies^[^ ^10^, ^13^ ^]^ and three were human studies.^[^ ^5^, ^12^, ^23^ ^]^ Six studies investigated the associations between folate status and sperm DNA damage and apoptosis, including two animal studies^[^ ^11^, ^13^ ^]^ and four human studies (**Table** **2**).^[^ ^7^, ^19^, ^21^, ^24^ ^]^

**Table 2:** Description and summary of data from 19 studies that investigated associations between folate and sperm quality and sperm epigenetics

Author	Year	Study type	Synthetic/natural folate	Sperm parameters	Sperm epigenetics	Sperm DNA damage	Sperm apoptosis
Lambrot et al.	2013	Animal study	Synthetic	=	+/−	−	=
Swayne et al.	2012	Animal study	Synthetic	=		−	
Ly et al.	2017	Animal study	Synthetic	+/−	+/−		
Yuan et al.	2017	Animal study and case‐control study	Synthetic Natural	+ +	=		
Murphy et al.	2011	Case‐control study	Natural	+			
Wallock et al.	2001	Case‐control study	Natural	+			
Boxmeer et al.	2007	Cross‐sectional study	Natural	=			
Boxmeer et al.	2009	Cross‐sectional study	Natural	−		−	
Crha et al.	2010	Cross‐sectional study	Natural	=			
Ebisch et al.	2006	Randomized controlled trial. Data used is cross‐sectional	Synthetic	=			
Ebisch et al.	2005	Randomized controlled trial	Synthetic				=
Boonyarangkul et al.	2015	Randomized controlled trial	Synthetic	+		−	
Da Silva et al.	2013	Randomized controlled trial	Synthetic	=			
Raigani et al.	2014	Randomized controlled trial	Synthetic	=		=	
Wong et al.	2002	Randomized controlled trial	Synthetic	=			
Chan et al.	2017	Retrospective intervention study	Synthetic		=		
Landau et al.	1978	Prospective intervention study	Synthetic	=			
Aarabi et al.	2015	Prospective intervention study	Synthetic	=	+/−	=	

+, positive association; −, negative association; =, no association.

#### 3.2.1 Sperm Parameters

##### Animal Studies

One study in mice comparing a 20‐fold FA fortified diet (40 mg kg^−1^) with a sevenfold FA deficient diet (0.3 mg kg^−1^), starting during pregnancy through maternal exposure and continued postnatally with a control diet (2 mg kg^−1^), found that both diets resulted in decreased sperm counts.^[^ ^10^ ^]^ One study showed that a folate deficient diet (0.3 mg kg^−1^) resulted in decreased sperm counts compared to a control (2 mg kg^−1^) diet (9.3 ± 1.2 × 10^6^ vs 13.0 ± 1.1 × 10^6^).^[^ ^12^ ^]^ Furthermore, Swayne et al. found no significant differences regarding sperm count when comparing a 6 mg kg^−1^ FA supplemented diet, starting during early developmental in utero until just after weaning, compared to a 2 mg kg^−1^ control diet (14.0 ± 1.5 × 10^6^ vs 13.0 ± 1.1 × 10^6^).^[^ ^11^ ^]^

Another study showed no significant difference in sperm count when mice received a folate deficient diet (0.3 mg kg^−1^ already started in utero through maternal exposure).^[^ ^13^ ^]^

In conclusion, animal studies show that both a FA supplemented and depleted diet can result in decreased sperm counts.

##### Human Studies

A total of five studies in human were designed as randomized controlled trials investigating the effect of FA supplement use on sperm parameters.^[^ ^6^, ^15^, ^16^, ^19^, ^21^ ^]^ Of these five RCTs, we could only use cross‐sectional data from one study for this systematic review.^[^ ^16^ ^]^ Three RCTs reported no significant differences regarding sperm volume, motility, and morphology in the FA supplement user group (all 5 mg FA per day) as compared to the control group.^[^ ^6^, ^15^, ^19^ ^]^ On the other hand, one of the RCTs showed a significant increase in sperm motility from 11.4% to 20.4% after 3 months of 5 mg per day FA supplement use.^[^ ^21^ ^]^ Only Raigani et al. showed that FA supplement use also caused a significant increase in serum FA from 4 ng mL^−1^ at baseline to 32.4 ng mL^−1^ after the intervention. Two non‐randomized intervention studies did not notice any effect on the same sperm parameters after a 30‐day trial of 10 mg FA supplementation and after 6 months of 5 mg FA supplementation.^[^ ^5^, ^17^ ^]^

The remaining seven human studies were either case‐control studies or cross‐sectional study designs.^[^ ^7^, ^12^, ^14^, ^16^, ^18^, ^20^, ^22^ ^]^ Four of these studies showed significant associations between FA supplement use and sperm parameters.^[^ ^7^, ^12^, ^18^, ^20^ ^]^ Wallock et al. showed that in healthy males, folate concentrations measured in seminal plasma (17.5 nmol L^−1^) correlated significantly with blood plasma folate (10.3 nmol L^−1^; *r* = 0.76*, p* < 0.001) and that seminal plasma folate significantly correlated with sperm density (*r* = 0.37*, p* < 0.05) and sperm total count (*r* = 0.31*, p* < 0.05).^[^ ^20^ ^]^ In line with this paper, Boxmeer et al. showed positive associations between seminal plasma folate (25.3 nmol L^−1^) and blood plasma folate (15.7 nmol L^−1^) in both fertile and subfertile men (*r* = 0.47*, p* < 0.001). Significant associations were found between blood folate concentrations and sperm parameters, although seminal plasma folate concentration was inversely correlated with ejaculate volume (*r* = −0.20*,p* < 0.01).^[^ ^7^ ^]^ One case‐control study showed that serum and red blood cell (RBC) folate concentrations were significantly lower in subfertile compared with fertile males (serum: 12.9 ± 5.9 and 14.7 ± 6.0 nmol L^−1^ (*p* = 0.006), respectively, and RBC: serum: 649.1 ± 203.6 and 714.5 ± 223.4 nmol L^−1^ (*p* = 0.044), respectively.^[^ ^18^ ^]^ In the logistic regression model, serum folate was a significant predictor of subfertility, especially among non‐users of vitamins (odds ratio 0.36 [95% confidence interval 0.16–0.78]). In addition, men with azoospermia showed significantly lower seminal plasma folate concentrations than men with normozoospermia (respectively, 24.0 nmol L^−1^ (interquartile range [IQR] 19.84–30.69) vs 26.2 nmol L^−1^ (IQR 21.7–34.8))^[^ ^12^ ^]^ and seminal plasma folate concentrations were significantly correlated with sperm density (*r* = 0.19*,p* < 0.01), but not with other sperm parameters.

The other three out of these seven studies did not find any associations between paternal folate status and sperm parameters.^[^ ^14^, ^16^, ^22^ ^]^ Chra et al. showed no significant differences regarding both blood and seminal plasma folate on sperm parameters, although in men with obstructive azoospermia, higher seminal plasma folate concentrations were found compared to non‐obstructive azoospermia (31.5 vs 20.7 nmol L^−1^, respectively).^[^ ^22^ ^]^ When comparing blood and seminal plasma folate concentrations between fertile and subfertile males, two studies found no significant associations with sperm parameters.^[^ ^7^, ^16^ ^]^

The quality of abovementioned studies according to the ErasmusAGE quality score ranged between 3 and 9, with the majority (54%) having a score above 7. Although, the RCTs were adequately designed according to the CONSORT statements,^[^ ^25^ ^]^ the number of included participants was low. Significantly positive associations were reported in the large case‐control studies of Yuan et al. (*n* = 269) and Murphy et al. (*n* = 337), whereas the smaller studies failed to show significance, which might be due to underpowerment. The study of Aarabi et al. was initiated to investigate effects on methylation status of the sperm, but without correcting for confounders. Of the remaining seven human studies, only two adjusted their statistical model for confounders to allow adequate interpretation of the results; the studies of Boxmeer et al. and Murphy et al. corrected for at least paternal age and smoking. It is important to take confounders into consideration since previous studies have shown that a diversity of conditions and factors, such as smoking, alcohol use, age, and BMI also influence sperm parameters, which is in line with the induction of excessive oxidative stress.^[^ ^26^, ^27^, ^28^, ^29^ ^]^ Although, less research is performed on paternal influences on pregnancy outcomes, we assume that the same confounding factors should be considered.

Only 7 out of 13 studies reported blood folate concentrations in the study population, ranging from 9 to 73 nmol L^−1^, while two reported concentrations before and after intervention. Unfortunately, the effects of normal values of folate concentrations regarding sperm quality are not mentioned. One might hypothesize that only men with low folate concentrations benefit from FA supplementation. This is supported by Murphy et al., who showed that an increase of folate from 13 to 25 nmol L^−1^ was associated with a significant increase in sperm parameters. However, the study of Raigani et al. found an increase from 9 to 73 nmol L^−1^ without a significant effect on sperm parameters.

##### Meta‐Analysis of Folic Acid Supplement Use and Sperm Parameters

**Figure 3:**
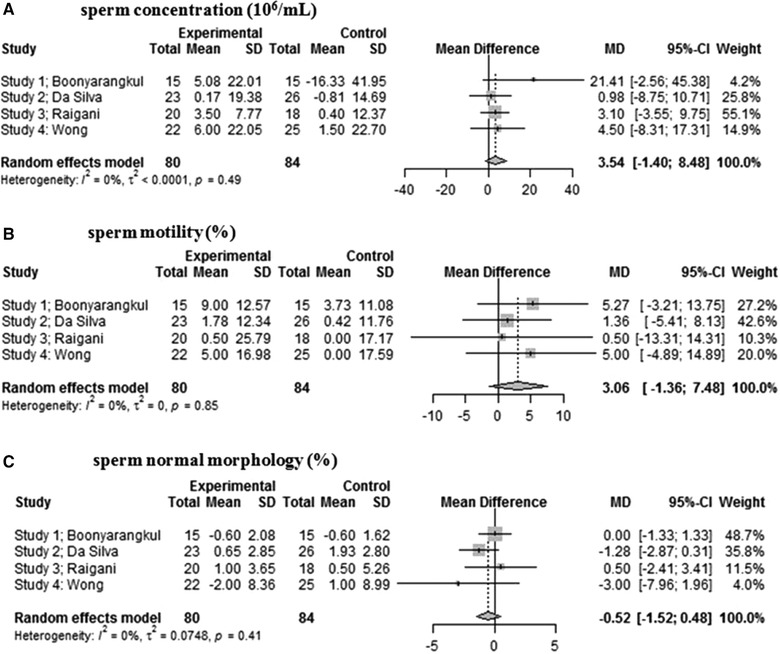
Forest plot of the effect of 5 mg folic acid supplement use in subfertile men: a) sperm concentration, b) sperm motility, and c) sperm morphology.

Four studies were eligible for a meta‐analysis to assess the combined effect of FA supplement use on sperm parameters in subfertile males.^[^ ^6^, ^15^, ^19^, ^21^ ^]^ Data of sperm concentration, motility, and normal morphology were, respectively, analyzed in a random‐effects model to estimate the effect of daily 5 mg FA treatment on each sperm parameter (**Figure** **3**). The results show that the sperm concentration was higher in patients after FA supplement use compared to control (3.54 95%CI [−1.40 to 8.48]); however, these results were not significantly different (*p* = 0.16). Sperm motility also did not significantly differ after FA supplement use compared to controls (3.06 95%CI [−1.36 to 7.48]) (*p* = 0.17). A non‐significant decrease after FA supplement use (−0.52 95%CI [−1.52 to 0.48]) was shown regarding sperm normal morphology (*p* = 0.31). There was no evidence of significant heterogeneity in the study populations regarding concentration, motility, and normal morphology (*I* ^2^ all 0%).

In conclusion, some human studies show associations between paternal folate status and sperm parameters. A meta‐analysis of four RCTs showed no significant differences regarding sperm parameters after 5 mg per day FA supplementation.

##### Discussion

Decreased folate concentrations alter the 1‐C metabolism resulting in a reduced availability of 1‐C groups and building blocks for DNA synthesis and repair, which are essential for successful spermatogenesis and genomic stability. Supporting this hypothesis, all non‐randomized controlled trials studies showed significant associations between folate concentrations and sperm parameters.^[^ ^7^, ^12^, ^18^, ^20^ ^]^ The suggestion that adequate folate concentrations could serve as protection against DNA damage is supported by an RCT showing a decrease in sperm DNA damage after 3 months of 5 mg per day FA supplement use.^[^ ^21^ ^]^ To compensate for a possible folate deficiency, FA supplement use will provide essential building blocks that could improve sperm quality parameters. Although, three out of the four RCTs did not find any significant improvements in sperm parameters after FA supplement use,^[^ ^6^, ^15^, ^19^ ^]^ Boonyarankul et al., showed a significant increase in percentage of sperm motility (11.40–20.40%) after 3 months of 5 mg per day FA supplement use. Three out of the four RCTs did not report whether folate concentrations in either blood or RBCs increased after the FA intervention, while Raigani et al. showed a significant increase in serum folate concentrations. Taking measurement of folate concentrations along, either in blood or RBCs, is especially interesting since there is heterogeneity in the data between before mentioned studies, which might be explained by either subjects not daily taking FA, differences in folate absorption, or in the conversion of dihydrofolate to tetrahydrofolate by intestinal dihydrofolate reductase.

In the additional meta‐analysis, we did notice a trend indicating that 3–6 months of daily FA treatment of 5 mg per day improves sperm volume and the percentage of sperm motility. The non‐significance of the meta‐analysis can be explained by the relatively small numbers of patients in each trial (*N* = 160 in total), since in studies in women around 6500 participants were needed to show significant effect on neural tube defects, for example.^[^ ^1^ ^]^

The relationship between low folate concentrations and sperm quality seems explainable; however, the possible detrimental effect of too high folate concentrations is less clear. It is hypothesized that excessive FA supplement use gives an increase in dihydrofolate, with a negative feedback signal on the MTHFR enzyme, thereby downregulating the biosynthesis of 1‐C groups. Without knowing the beneficial and detrimental effects of exposure to different concentrations of FA, we should be cautious with the global administration of high doses of FA due to possible teratogenicity.^[^ ^30^ ^]^

#### 3.2.2 Sperm DNA Damage and Methylation

##### Animal

Sperm DNA damage was investigated in two independent studies in mice, showing that a folate deficient diet results in increased DNA damage.^[^ ^11^, ^13^ ^]^ Lambrot et al. showed an increase in the expression of a histone variant (γH2AX) involved in repair of DNA double strand breaks, while the total number of DNA double strand breaks in spermatocytes remained comparable between the groups indicating that DNA damage was correctly repaired.^[^ ^13^ ^]^

Another way to measure DNA damage is via the DNA fragmentation index (DFI), where a higher DFI indicates more DNA damage. Swayne et al. showed that mice weaned to a folate deficient diet (see Table 1 for exact folic acid concentrations) had an increased percentage DFI, compared to a control diet (5.0% ± 0.9 vs 2.6% ± 0.1, *p* = 0.04).^[^ ^11^ ^]^ Furthermore, two other animal studies showed that a low intake of dietary folate resulted in increased as well as decreased sperm DNA‐methylation.^[^ ^10^, ^13^ ^]^ Lambrot et al. showed that DNA methylation concentrations in general were both increased and decreased for various genes in the folate deficient group as compared to the control, while histone methylation was primarily downregulated. No differences regarding sperm apoptosis or methylation status of imprinted genes were reported.^[^ ^13^ ^]^ Ly et al. showed that both a high FA supplemented and depleted diet resulted in increased variance in methylation across imprinted genes, required for normal fetal development.^[^ ^10^ ^]^

In conclusion, in animal models, an FA depleted diet results in more sperm DNA damage and both increased and decreased sperm DNA methylation.

##### Human

Sperm DNA damage was reported by four studies.^[^ ^5^, ^7^, ^19^, ^21^ ^]^ Two RCTs reported a decrease in sperm DNA damage after FA treatment.^[^ ^19^, ^21^ ^]^ Boonyarangkul et al. showed a significant decrease in DNA tail length based on a Comet assay, indicating less DNA damage, from 14.59 µm to 4.04 µm (*p* <.05) after 3 months of 5 mg per day FA treatment.^[^ ^21^ ^]^ Raigani et al. showed a non‐significant decrease in DFI after a 16‐week 5 mg per day FA intervention compared to placebo (from 31.7 ± 14.8% to 24.3 ± 12% vs from 34.5 ± 19.7% to 29.5 ± 10%, respectively).^[^ ^19^ ^]^ Same results were shown in the Aarabi study. One cross‐sectional study showed that only in the fertile male group, the seminal plasma folate concentrations were negatively associated with DFI (*r* = −0.36, *p* < 0.05).^[^ ^7^ ^]^

Annexin A5 (AnxA5) is commonly used to detect apoptotic cells by its ability to bind to phosphatidylserine, a marker of apoptosis, which presents on the exterior part of the plasma membrane. Hence, a high concentration of AnxA5 indicates high concentrations of apoptosis. Seminal AnxA5 was determined in one RCT to assess the effect of FA supplement use on seminal apoptosis. After a 26 week period of 5 mg per day FA intervention there was a slight decrease in AnxA5 in both fertile (from 5.6 to 5.4 µg mL^−1^) and subfertile (from 5.4 to 5.2 µg mL^−1^) males; however, it failed to reach significance.^[^ ^24^ ^]^

A total of three studies investigated the effect of folate status on sperm DNA methylation.^[^ ^5^, ^12^, ^23^ ^]^ The recent study of Chan et al. showed that multiple years of FA food fortification in Canada had no significant influence on sperm overall DNA methylation.^[^ ^23^ ^]^ However, 6 months of 5 mg per day FA supplement use caused genome wide hypomethylation and hypermethylation covering intergenic regions, introns, and exons of the sperm DNA.^[^ ^5^ ^]^ This effect is aggravated in individuals who are homozygous for the *MTHFR 677C>T* polymorphism. Aarabi et al. found no effect of FA supplement use on the differentially methylated regions of several imprinted genes.^[^ ^5^ ^]^ A case‐control study showed no differences in methylation pattern of the promotor regions of some spermatogenesis key‐genes (*Esr1, Cav1*, and *Elavl1*) in males with low versus high seminal plasma and blood folate concentrations.^[^ ^12^ ^]^

The articles reporting on DNA damage were of good quality (75% received a quality score of 8 or higher), whereas all studies investigating methylation of sperm were of low quality (66% received a quality score of 4 or lower). Concerning potential confounders in these studies, two studies are designed as RCTs in which correction for confounders is not needed. Boxmeer et al. correctly adjusted for several confounding factors such as age, BMI, smoking, and alcohol use, while the Aarabi et al. study did not apply any correction for confounders.

In conclusion, in humans, multiple studies show that FA supplementation results in a reduction in sperm DNA damage with some studies showing that folate status is associated with the sperm epigenome.

##### Discussion

Chronic exposure to high dose synthetic FA and low folate concentrations seems to induce excessive oxidative stress and as such cause increased cellular apoptosis and seminal DNA damage. Techniques used to measure sperm DNA damage included the sperm chromatin structure assay (SCSA; two studies), the comet assay (one study), and acridine orange staining (AO‐test; one study). While the SCSA and comet assay are both sensitive and reliable, the AO‐test appears to have a relative lack of reproducibility.^[^ ^31^ ^]^ A recent guideline regarding DNA damage does not recommend using a specific technique, but mentions that SCSA is one of the most used techniques. The low number of studies (four in total) and the usage of different tests, do not allow comparison between studies.

Folate is as substrate involved in the synthesis of lipids, proteins, DNA, and RNA, the scavenging of reactive oxidative radicals, DNA repair, and epigenetic. These mechanisms are involved in cell multiplication and cell differentiation, apoptosis, signaling, and programming and as such in spermatogenesis and embryogenesis. Elevated levels of reactive oxygen species (ROS), caused by various chronic diseases, obesity, genetic variations, medication use, ageing, and an unhealthy diet and lifestyle, will lead to oxidative stress, which is an important cause of DNA damage. A crucial function of the 1‐C cycle is scavenging of these reactive oxygen species (ROS) by the anti‐oxidant glutathione, which is synthesized from folate together with homocysteine. Importantly, an unhealthy diet is associated with a decreased intake of folate. Only when concentrations of methionine and especially folate are sufficient, glutathione is formed. Low intake of FA and folate are associated with an increase in oxidative stress thereby altering DNA‐integrity and subsequent molecular processes involved in spermatogenesis and embryogenesis. The studies in this review show that FA supplement use can result in decreased sperm DNA damage.

### 3.3 Postconceptional

Seven articles reported on associations between paternal folate status and the post‐conceptional outcomes, such as fertility, embryonic growth, miscarriage, fetal development, congenital malformations, placentation, and pregnancy outcomes, of which five were animal studies ^[^ ^10^, ^13^, ^32^, ^33^, ^34^ ^]^ and two were human studies (**Table** **3**).^[^ ^35^, ^36^ ^]^

**Table 3:** Description and summary of data from nine studies that investigated associations between folate and postconceptional outcomes

Author	Year	Study type	Synthetic/natural folate	Fertility	Miscarriage	Birthweight	Fetal liver	Fetal brain	Placenta	Congenital malformations
Kim et al.	2011	Animal study	Synthetic			=	+		+	
Kim et al.	2013	Animal study	Synthetic			+	+	+		
Lambrot et al.	2013	Animal study	Synthetic	+	−	=			=	−
Ly et al.	2017	Animal study	Synthetic		+	=		+	=	=
Mejos et al.	2013	Animal study	Synthetic			=	+			
Pauwels et al.	2017	Prospective cohort study	Natural			=				=
Ratan et al.	2008	Case‐control study	Natural							−

+, positive association; −, negative association; =, no association.

#### 3.3.1 Fertility

##### Animal

Only one animal study showed that a folate deficient diet in mice resulted in decreased pregnancy rates compared to mice fed control diet (52.4% and 85.0%, respectively).^[^ ^13^ ^]^

##### Human

There are no human studies reporting on fertility in relation to paternal folate status.

##### Discussion

The overall results of the selected articles in this review show that paternal folate status is often positively associated with sperm parameters. The sperm parameters concentration and percentage of mobile sperm are associated with fertility and ongoing pregnancy rates.^[^ ^37^, ^38^ ^]^ Therefore, the reasoning is that in future, fathers’ optimization of folate status has the potential to beneficially influence male fertility and pregnancy chances of a couple. Unfortunately, until now no human studies have shown any effect of the paternal folate status on pregnancy‐chance. However, strong adherence of a couple to a diet very rich in natural folate, like the Mediterranean diet, increases the chance of an ongoing pregnancy after an IVF/ICSI treatment.^[^ ^39^, ^40^ ^]^

#### 3.3.2 Embryonic Growth and Development

##### Animal

Two animal studies in mice investigated the association between the paternal FA supplement use and embryonic growth and miscarriages.^[^ ^10^, ^13^ ^]^ Lambrot et al. showed that the offspring of male mice, which had received a folate deficient diet from early embryonic development onward (0.3 mg FA per kg), did not differ regarding embryonic weight and crown rump length (CRL) compared to male mice on a control diet (2 mg kg^−1^). However, they found that a paternal folate deficient diet resulted in a twofold increase of post‐implantation embryonic loss in mice.^[^ **^13^** ^]^ Another animal study showed that male mice fed a highly FA fortified diet (40 mg kg^−1^) have an increased risk of post‐implantation embryonic loss and their offspring show growth restriction compared to control diet (2 mg kg^−1^).^[^ ^10^ ^]^ In conclusion, in animals, both very high and very low FA intake is associated with an increased miscarriage rate.

##### Human

There are no human studies reporting on the association between paternal folate status and embryonic growth and development or miscarriage.

##### Discussion

Shortly after conception, a global loss of methylation at the level of DNA and histones takes place (Figure 1a).^[^ ^41^ ^]^ However both paternally and maternally imprinted genes, such as insulin like growth factor (*IGF‐2*), are unaffected by this demethylation wave.^[^ ^42^ ^]^ Since imprinting of these genes occurs during the process of male and female gametogenesis, studying the effects of periconceptional lifestyle factors on embryonic health and health later in life, makes these genes of special interest. Imprinted genes have a parent‐of‐origin effect by preferential expression of either maternal or paternal inherited allele and emphasize the parental influence during the periconception period.

Altered sperm DNA methylation in genes for normal growth and development of embryonic growth and development could be affected by epigenetic imprinting.

#### 3.3.3 Fetal Liver and Brain

##### Animal

The insulin‐like‐growth factor 2 (*IGF‐2*) gene is paternally expressed and encodes for a protein that plays a major role in regulating embryonic growth and development.^[^ ^43^ ^]^ Three animal studies investigated the effect of a paternal folate deficiency on fetal liver outcomes.^[^ ^32^, ^33^, ^34^ ^]^ All studies showed that the fetal liver folate content was decreased after a paternal folate deficient diet compared to control diet. Mejos et al. showed that a folate deficient diet significantly decreased global hepatic DNA‐methylation concentrations with 37.9%, although no significant differences in hepatic IGF‐2 expression when compared to a folate sufficient diet were detected.^[^ ^34^ ^]^

Two other animal studies investigated the association between paternal folate status and brain development.^[^ ^10^, ^33^ ^]^ One study showed that the total folate content of the fetal brain was comparable in rats on a folate deficient and control diet, whereas the IGF‐2 protein expression in the fetal whole brain was decreased in former group.^[^ ^33^ ^]^ Interestingly, they also found a significant decrease in whole brain DNA‐methylation, as measured by the quantity of 5‐methylcytosine (5‐MC). The percentage of 5‐MC decreases from 4.5% to 2.6% when comparing a folate sufficient diet with a folate deficient diet. Another animal study that investigated global brain methylation failed to see an effect of a paternal high or low folate diet.^[^ ^10^ ^]^ They did, however, find a significant increase in variance of DNA methylation on a locus of the paternally expressed gene 1 (*PEG1*) in the group supplemented with high FA.

In conclusion, in animals all studies show an effect of paternal folate diets on fetal liver contents while some indicate effects on diverse fetal brain measurements.

##### Human

There are no human studies reporting on the fetal development of brain and liver.

#### 3.3.4 Congenital Anomalies

##### Animal

Two animal studies investigated the association between paternal folate status and congenital anomalies, of which one found an association between a FA deficient diet compared to control mice.^[^ ^13^ ^]^ Lambrot et al. showed that in fathers on a folate deficient diet, the percentage of litters with congenital malformations is significantly increased when compared to a control diet (27% and 3%). The abnormalities consisted of craniofacial abnormalities, limb defects, muscle and skeletal malformations.^[^ ^13^ ^]^ Another study found no significant differences between male mice on a control diet compared to those on high FA or low FA diet.^[^ ^10^ ^]^

In conclusion, in animals some studies show a negative association between FA intake and congenital malformations.

##### Human

One human study investigated the association between paternal folate status and congenital malformations.^[^ ^36^ ^]^ They found that the fathers of children born with neural tube defects had significantly lower folate concentrations compared to fathers of children born with other or without congenital malformations. Although, they reported an odds ratio for neural tube defects of 5.2 (95%CI: 1.3–20.8) of offspring of fathers with low folate concentrations, the effect diminished when adjusting for potential confounders, which were unfortunately not mentioned.

In conclusion, in humans only one study showed a negative association between folate intake and congenital malformations.

##### Discussion

Low intake of folate and low folate concentration are associated with increased sperm DNA damage and alterations in sperm epigenetics, which in case of successful fertilization could interfere with embryonic development. The human study underlines that paternal folate status can effect embryonic growth, most likely by altering the sperm epigenome and thereby inducing adverse pregnancy outcomes.^[^ ^36^ ^]^ However, results need to be interpreted with caution since residual confounding cannot be excluded due to the lack of mentioning of adjusted confounders (ErasmusAGE quality score of 3).

The number of women needed to use FA supplements periconceptionally to prevent one child with a neural tube defect is 847 (NNT = 847).^[^ ^44^ ^]^ For men, this number is most likely much higher, since FA use by women directly affect the intrauterine environment and could potentially compensate for or correct paternal effects of folate deficiencies. Males most likely pass on folate effects via sperm DNA methylation changes and concentration in seminal fluid.^[^ ^45^ ^]^ More diverse and intensive human research is necessary before we can translate the results of the mouse models to humans.

#### 3.3.5 Placentation

##### Animal

Three animal studies describe the effect of paternal folate on general aspects of placentation, such as weight, size, and folate content.^[^ ^10^, ^13^, ^32^ ^]^ Two studies did not find any significant differences when comparing placenta weight and size between a paternal FA deficient and control diet (see Table 1 for exact folic acid concentrations).^[^ ^10^, ^13^ ^]^ Another study found a lower placental weight and a lower total placental folate content in the folate deficient diet group compared to control.^[^ ^32^ ^]^ Surprisingly, Lambrot et al. reported two fused placentas, which is considered to be abnormal, out of the group of 35 pregnancies.

Regarding the methylation status of the placenta, one study found no significant differences in global placental methylation concentrations when comparing both low and high FA paternal content diets compared to control.^[^ ^10^ ^]^ However, in the group with very high FA fortified diets (folate concentration 20 times higher as compared to the control) compared to control diet, inter‐individual alterations in methylation across the paternally expressed genes small nuclear ribonucleoprotein polypeptide N and paternally expressed gene 3 were found.

Placental transporter proteins are necessary and essential for the transport of micronutrients over the placental barrier. Of these proteins, the placental folate receptor alpha enzyme is crucial for the transport of folate over the placenta. Interestingly, Kim et al. showed that a paternal folate deficient diet resulted in a significant upregulation of this enzyme expression compared to wildtype rats (2.3 times higher expression).

In conclusion, in animals some studies show a negative association between paternal FA intake and placenta weight and development and an association with alterations in placenta epigenetics.

##### Human

There are no human studies reporting on associations between paternal folate status and placental development.

##### Discussion

Paternally imprinted genes, which in general are excluded from the postconceptional de‐ and remethylation wave, are predominantly expressed in the placenta (Figure 1a). External influences such as nutrition, lifestyle, and folate status throughout the preconception stage can influence the definitive epigenetic programming of (imprinted) genes, with potential negative effects on embryonic but also placental development postconceptionally. Micronutrients like iron, vitamin D, vitamin A, folate, and vitamin B12 are necessary for normal placental development. Deficiencies of these micronutrients in women are associated with impaired placental development, which is associated with negative pregnancy outcomes.^[^ ^46^ ^]^ In women, FA supplement use is also associated with placental development, since placental weight at birth between women using FA supplements versus women not using FA supplements is different (643 grams vs 626 grams, respectively).^[^ ^47^ ^]^ The causal effect of these paternal factors remains to be elucidated, but epigenetic programming of paternal origin is a plausible mechanism.

#### 3.3.6 Pregnancy Outcome

##### Animal

Five animal studies investigated the association between paternal folate status and birthweight.^[^ ^10^, ^13^, ^32^, ^33^, ^34^ ^]^ Four studies did not find an association between a folate deficient diet and birthweight,^[^ ^10^, ^13^, ^32^, ^34^ ^]^ whereas a very high FA fortified diet also did not alter birthweight compared to controls.^[^ ^10^ ^]^ One animal study, however, showed that a folate deficient diet compared to control diet resulted in lower birthweight (2.1–2.3 grams [*p* < 0.001]) and smaller crown rump length (CRL) (3.3–3.4 cm [*p* < 0.05]).^[^ ^33^ ^]^ Interestingly, one study found an increase in postnatal deaths when comparing both very high and low FA fortified diets compared to control mice.^[^ ^10^ ^]^

In conclusion, in animals, a minority of studies showed an association between paternal folate diet and pregnancy outcomes.

##### Human

There is one human study (ErasmusAGE quality score of 8) reporting on birthweight, which found no significant association between paternal folate intake, as measured by food questionnaires, and birthweight of the offspring.^[^ ^35^ ^]^

## 4 Strengths and Limitations

The present work is the first to systematically review the currently available evidence on the impact of the paternal folate status on male fertility factors from sperm quality to pregnancy outcomes. Due to the lack of human studies on paternal effects of FA supplement use, we included animal studies to gain more insight into the (patho)physiologic mechanisms and the (epi)genetic effects of FA supplement use, resulting in a translational systematic review. The review also includes an additionally performed meta‐analysis on the associations between paternal FA supplement use ranging from 3 to 6 months and sperm parameters concentration, motility, and normal morphology. Despite our extensive literature search, the amount of evidence and quality of the studies was relatively low. Regarding the included animal studies; in a number of studies, the FA intervention already started in utero, during the key time of parental erasure and reprogramming of the germ cell epigenome and continuing postnatally for varying amounts of time. The extended exposure might have lifelong effects on the male germ cell, perturbing prenatal and postnatal germ cell development and epigenetics. Nevertheless, this review provides some evidence that the periconceptional paternal folate status or diet, can influence sperm parameters, fertility, embryonic growth, and pregnancy outcomes possibly explained via an impaired embryonic and/or placental DNA synthesis and repair, epigenetic programming, or cell multiplication.

Unfortunately, optimal ranges of folate concentrations in males are lacking in both human and animals, making comparisons between studies difficult. Several study results indicate that either too low or too high concentrations are not beneficial. Before any general recommendations for paternal FA supplement use can be issued, further investigation is necessary to better understand the contribution of the paternal folate status on fertility and pregnancy outcomes, including placentation.

## 5 Conclusion

This translational systematic review shows that the paternal folate status in humans and animals might be associated with sperm quality and subsequent pregnancy outcomes, like fetal development, placentation, and congenital malformations. As in women, not only low but also high folate concentrations are associated with negative outcomes in men, such as poorer sperm quality and an increased risk of congenital malformations. In general, low paternal folate status is associated with poorer outcomes, while deficiencies can easily be supplemented with FA tablets and fortified diets. However, in recent years, the concerns of high folate concentrations are increasing,^[^ ^30^ ^]^ especially with the worldwide increase of the use of multivitamin supplements and FA fortified foods. Therefore, we have to be increasingly aware of also the risk of harmful effects of too high (supplementary) folate concentrations in developed countries. Furthermore, the results of this systematic review make it clear that human data on paternal folate status and fertility and pregnancy outcome is very scarce. More research is necessary into the periconceptional roles of paternal micronutrients. We need to understand the effects of paternal folate status on sperm epigenome and periconception outcomes, so we can optimally counsel future parents during the periconception period.

## Conflict of Interest

The authors declare no conflict of interest.

## Supporting information

Supporting Information

## References

[1] L. M. De‐Regil , J. P. Pena‐Rosas , A. C. Fernandez‐Gaxiola , P. Rayco‐Solon , Cochrane Database Syst. Rev. 2015, 12, CD007950. 10.1002/14651858.CD007950.pub3 PMC8783750 26662928

[2] E. M. van Uitert , R. P. Steegers‐Theunissen , Mol. Nutr. Food Res. 2013, 57, 582. 23213022 10.1002/mnfr.201200084

[3] R. P. Steegers‐Theunissen , J. Twigt , V. Pestinger , K. D. Sinclair , Hum. Reprod. Update 2013, 19, 640. 23959022 10.1093/humupd/dmt041

[4] S. J. Mentch , J. W. Locasale , Ann. N. Y. Acad. Sci. 2016, 1363, 91. 26647078 10.1111/nyas.12956 PMC4801744

[5] M. Aarabi , M. C. San Gabriel , D. Chan , N. A. Behan , M. Caron , T. Pastinen , G. Bourque , A. J. MacFarlane , A. Zini , J. Trasler . Hum. Mol. Genet. 2015, 24, 6301. 26307085 10.1093/hmg/ddv338 PMC4614702

[6] W. Y. Wong , H. M. W. M. Merkus , C. M. G. Thomas , R. Menkveld , G. A. Zielhuis , R. P. M. Steegers‐Theunissen , Fertil. Steril. 2002, 77, 491. 11872201 10.1016/s0015-0282(01)03229-0

[7] J. C. Boxmeer , M. Smit , E. Utomo , J. C. Romijn , M. J. C. Eijkemans , J. Lindemans , J. S. E. Laven , N. S. Macklon , E. A. P. Steegers , R. P. M. Steegers‐Theunissen , Fertil. Steril. 2009, 92, 548. 18722602 10.1016/j.fertnstert.2008.06.010

[8] C. Rathke , W. M. Baarends , S. Awe , R. Renkawitz‐Pohl , Biochim. Biophys. Acta 2014, 1839, 155. 24091090 10.1016/j.bbagrm.2013.08.004

[9] O. Hamilton , *Quality Assessment Tool for Quantitative Studies*, National Collaborating Centre for Methods and Tools, Hamilton, Ontario 2008 http://dev.nccmt.ca/resources/search/14

[10] L. Ly , D. Chan , M. Aarabi , M. Landry , N. A. Behan , A. J. MacFarlane , J. Trasler , Mol. Hum. Reprod. 2017, 23, 461. 28535307 10.1093/molehr/gax029 PMC5909862

[11] B. Swayne , A. Kawata , N. Behan , A. Williams , M. G. Wade , A. J. Macfarlane , C. L. Yauk , Mutat. Res. 2012, 737, 1. 22824165 10.1016/j.mrfmmm.2012.07.002

[12] H. F. Yuan , K. Zhao , Y. Zang , C. Y. Liu , Z. Y. Hu , J. J. Wei , T. Zhou , Y. Li , H. P. Zhang , Oncotarget 2017, 8, 24130. 28445960 10.18632/oncotarget.15731 PMC5421833

[13] R. Lambrot , C. Xu , S. Saint‐Phar , G. Chountalos , T. Cohen , M. Paquet , M. Suderman , M. Hallett , S. Kimmins , Nat. Commun. 2013, 4, 2889. 24326934 10.1038/ncomms3889 PMC3863903

[14] J. Boxmeer , M. Smit , R. Weber , J. Lindemans , J. C. Romijn , M. J. Eijkemans , N. S. Macklon , R. P. Steegers‐Theunissen , J. Androl. 2007, 28, 521. 17287458 10.2164/jandrol.106.001982

[15] T. M. da Silva , M. C. S. Maia , J. T. Arruda , F. C. Approbato , C. R. Mendonça , M. S. Approbato , J. Bras. Reproducao. Assistida 2013, 17, 152.

[16] I. M. W. Ebisch , F. H. Pierik , F. H. De Jong , C. M. G. Thomas , R. P. M. Steegers‐Theunissen , Int. J. Androl. 2006, 29, 339. 16533356 10.1111/j.1365-2605.2005.00598.x

[17] B. Landau , R. Singer , T. Klein , E. Segenreich , Experientia 1978, 34, 1301. 570119 10.1007/BF01981432

[18] L. E. Murphy , J. L. Mills , A. M. Molloy , C. Qian , T. C. Carter , H. Strevens , D. Wide‐Swensson , A. Giwercman , R. J. Levine , Asian J. Androl. 2011, 13, 856. 21857689 10.1038/aja.2011.96 PMC3372894

[19] M. Raigani , B. Yaghmaei , N. Amirjannti , N. Lakpour , M. M. Akhondi , H. Zeraati , M. Hajihosseinal , M. R. Sadeghi , Andrologia 2014, 46, 956. 24147895 10.1111/and.12180

[20] L. M. Wallock , T. Tamura , C. A. Mayr , K. E. Johnston , B. N. Ames , R. A. Jacob , Fertil. Steril. 2001, 75, 252. 11172823 10.1016/s0015-0282(00)01697-6

[21] A. Boonyarangkul , N. Vinayanuvattikhun , C. Chiamchanya , P. Visutakul , J. Med. Assoc. Thai. 2015, 98, 1057. 26817175

[22] I. Crha , M. Kralikova , J. Melounova , P. Ventruba , J. Zakova , R. Beharka , R. Husicka , M. Pohanka , M. Huser , J. Assist. Reprod. Gen. 2010, 27, 533. 10.1007/s10815-010-9458-8 PMC2965344 20676751

[23] D. Chan , S. McGraw , K. Klein , L. M. Wallock , C. Konermann , C. Plass , P. Chan , B. Robaire , R. A. Jacob , C. M. T. Greenwood , J. M. Trasler , Hum. Reprod. 2017, 32, 272. 27994001 10.1093/humrep/dew308 PMC5260859

[24] I. M. W. Ebisch , W. L. Van Heerde , C. M. G. Thomas , S. C. M. Schoormans , R. P. M. Steegers‐Theunissen , Annexins 2005, 2, e13.

[25] D. Rennie , J. Am. Med. Assoc. 2001, 285, 2006.

[26] D. Guo , W. Wu , Q. Tang , S. Qiao , Y. Chen , M. Chen , M. Teng , C. Lu , H. Ding , Y. Xia , L. Hu , D. Chen , J. Sha , X. Wang , Oncotarget 2017, 8, 48619. 28159940 10.18632/oncotarget.14950 PMC5564712

[27] R. M. Mostafa , Y. S. Nasrallah , M. M. Hassan , A. F. Farrag , A. Majzoub , A. Agarwal , Andrologia 2018, 50, e12910. 10.1111/and.12910 29124782

[28] E. Ricci , S. Al Beitawi , S. Cipriani , M. Candiani , F. Chiaffarino , P. Vigano , S. Noli , F. Parazzini , Reprod. BioMed. Online 2017, 34, 38. 28029592 10.1016/j.rbmo.2016.09.012

[29] A. Salas‐Huetos , M. Bullo , J. Salas‐Salvado , Human Reprod. Update 2017, 23, 371. 10.1093/humupd/dmx006 28333357

[30] A. D. Smith , Y. I. Kim , H. Refsum , Am. J. Clin. Nutr. 2008, 87, 517. 18326588 10.1093/ajcn/87.3.517

[31] A. Agarwal , A. Majzoub , S. C. Esteves , E. Ko , R. Ramasamy , A. Zini , Transl. Androl. Urol. 2016, 5, 935. 28078226 10.21037/tau.2016.10.03 PMC5182232

[32] H. Kim , Y. Choi , K. Kim , T. Tamura , N. Chang , Nutr. Res. Pract. 2011, 5, 112. 21556224 10.4162/nrp.2011.5.2.112 PMC3085799

[33] H. Kim , K. Kim , Y. Choi , N. Chang , Molecul. Nutr. Food 2013, 57, 671. 10.1002/mnfr.201200558 23229416

[34] K. K. Mejos , H. W. Kim , E. M. Lim , N. Chang , Nutr. Res. Pract. 2013, 7, 281. 23964315 10.4162/nrp.2013.7.4.281 PMC3746162

[35] S. Pauwels , I. Truijen , M. Ghosh , R. C. Duca , S. A. S. Langie , B. Bekaert , K. Freson , I. Huybrechts , G. Koppen , R. Devlieger , L. Godderis , J. Dev. Origins Health Dis. 2017, 8, 311. 10.1017/S2040174417000046 28260562

[36] S. K. Ratan , K. N. Rattan , R. M. Pandey , S. Singhal , S. Kharab , M. Bala , V. Singh , A. Jhanwar , Pediatr. Surg. Int. 2008, 24, 803. 18463884 10.1007/s00383-008-2167-z

[37] K. Madbouly , A. Isa , M. Habous , R. Almannie , B. Abu‐Rafea , S. Binsaleh , Can. J. Urol. 2017, 24, 8847. 28646941

[38] J. A. Hamilton , M. Cissen , M. Brandes , J. M. Smeenk , J. P. de Bruin , J. A. Kremer , W. L. Nelen , C. J. Hamilton , Human Reprod. 2015, 30, 1110. 10.1093/humrep/dev058 25788568

[39] M. Vujkovic , J. H. de Vries , J. Lindemans , N. S. Macklon , P. J. van der Spek , E. A. Steegers , R. P. Steegers‐Theunissen , Fertil. Steril. 2010, 94, 2096. 20189169 10.1016/j.fertnstert.2009.12.079

[40] J. M. Twigt , M. E. Bolhuis , E. A. Steegers , F. Hammiche , W. G. van Inzen , J. S. Laven , R. P. Steegers‐Theunissen , Human Reprod. 2012, 27, 2526. 10.1093/humrep/des157 22593431

[41] W. Reik , W. Dean , J. Walter , Science 2001, 293, 1089. 11498579 10.1126/science.1063443

[42] M. S. Bartolomei , Genes Dev. 2009, 23, 2124. 19759261 10.1101/gad.1841409 PMC2751984

[43] D. Bergman , M. Halje , M. Nordin , W. Engstrom , Gerontology 2013, 59, 240. 23257688 10.1159/000343995

[44] J. Lumley , L. Watson , M. Watson , C. Bower , Cochrane Database Syst. Rev. 2001, 3, CD001056. 10.1002/14651858.CD001056 11686974

[45] S. A. Robertson , D. J. Sharkey , Fertil. Steril. 2016, 106, 511. 27485480 10.1016/j.fertnstert.2016.07.1101

[46] B. C. Baker , D. J. Hayes , R. L. Jones , Reproduction 2018, 156, R69. 29844225 10.1530/REP-18-0130

[47] S. Timmermans , V. W. Jaddoe , L. M. Silva , A. Hofman , H. Raat , R. P. Steegers‐Theunissen , E. A. Steegers , Nutr., Metab. Cardiovasc. Dis. 2011, 21, 54. 19819678 10.1016/j.numecd.2009.07.002

