# Supplementary material for: Paternal Folate Status and Sperm Quality, Pregnancy Outcomes, and Epigenetics: A Systematic Review and Meta‐Analysis
Source: Mol Nutr Food Res. 2020 Feb 20;64(9):1900696. doi: 10.1002/mnfr.201900696 (PMC7317557; doi:10.1002/mnfr.201900696)
Supplement: Supplementary file 1 — Supporting Information [file MNFR-64-1900696-s001.docx]

**Supporting Information**

**Supplemental Table 1**. ErasmusAGE quality score form for systematic reviews adjusted for:

The effect of preconceptional paternal folate status on sperm quality, epigenetics and pregnancy outcomes; a systematic review.

*Original: ErasmusAGE, 24 June 2013*

This quality score can be used to assess the quality of studies included in systematic reviews and meta-analyses and is applicable to both interventional and observational studies. The score was designed based on previously published scoring systems (Carter et al, 2010 and the Quality Assessment Tool for Quantitative Studies*)*. The quality score is composed of 5 items, and each item is allocated 0, 1 or 2 points. This allows a total score between 0 and 10 points, 10 representing the highest quality.

The version presented below is a general version and needs to be adapted for each review separately, e.g. concerning what study size is large or small within the study field, what exposure and outcome measurement methods are adequate, and what the key confounders are. Decisions on these detailed criteria should be based on literature, guidelines and/or discussions with experts. The criteria should be defined before the review process.

1. **Study design**

**0** for studies with cross-sectional data collection

**1** for studies with longitudinal data collection (both retrospective and prospective)

**2** for intervention studies

1. **Study size** (predefined)*

**0** small population for analysis ( <50 patients)

**1** intermediate population for analysis (50-150 patients)

**2** large population for analysis (>150 patients)

1. **Exposure**

*Observational studies*

**0** if the study used no appropriate exposure measurement method or if not reported

**1** if the study used moderate quality exposure measurement methods

**2** if the study used adequate exposure measurement methods

*Intervention studies*

**0** if the intervention was not described or not blinded

**1** if the intervention was adequately single blinded.

**2** if the intervention was adequately double-blinded.

1. **Outcome**

**0** if the study used no appropriate outcome measurement method or if not reported

**1** if the study used moderate quality outcome measurement methods

**2** if the study used adequate outcome measurement methods ( Sperm quality according to WHO, sperm epigenetics, miscarriage, preterm birth, small for gestational age, fetal abnormalities)

1. **Adjustments** †*

**0** if findings are not controlled for at least key confounders (BMI, smoking, alcohol, age)

**1** if findings are controlled for key confounders (BMI, smoking, alcohol, age)

**2** if findings are additionally controlled for additional covariates or when an intervention is adequately randomized

* Needs to be specified for each review, based on literature, guidelines and/or expert opinions in the field

† Either adjusted for in the statistical analyses; stratified for in the analyses; or not applicable (e.g. a study in women only does not require controlling for sex)

**Supplemental Table 2**. Key search terms

| **Keywords** |  |  |
| --- | --- | --- |
| **EXPOSURE** | **OUTCOME** | |
| Folic acid | **Preconceptional** | **Postconceptional** |
| Folate | Sperm concentration | Fertility |
| MTHFR | Sperm quality | Male infertility |
| Vitamins | Sperm | Male fertility |
| Diet | Sperm dysfunction | Time to pregnancy |
| Antioxidants | Sperm count | Implantation |
| One carbon metabolism | VCM | Pregnancy |
| Homocysteine | Seminal plasma | Fetal growth |
| Metabolome | Ejaculate volume | Placenta |
|  | Asthenozoospermia | Placentation |
|  | Oligozoospermia | Pregnancy outcome |
|  | Oligoasthenozoospermia | Life birth ratio |
|  | Teratozoospermia | Miscarriage |
|  | Epigenetics | Congenital malformations |
|  | DNA methylation | Fetal growth retardation |
|  | DNA damage | Fetal growth restriction |
|  | Histone modifications | Small for Gestational Age |
|  |  | Intrauterine growth restriction |
|  |  | Premature birth |
|  |  | Preterm birth |
|  |  | Intrauterine death |
